# Wondering PTCA Wire: Retrieval by Tangling Technique

**DOI:** 10.14740/cr392w

**Published:** 2015-04-06

**Authors:** Santosh Kumar Sinha, Chandra Mohan Verma, Vinay Krishna, Ramesh Thakur, Prakash Kumar, Amit Goel, Ashutosh Kumar, Mahmadulla Razi

**Affiliations:** a Department of Cardiology, LPS Institute of Cardiology, G.S.V.M. Medical College, G. T. Road, Kanpur, Uttar Pradesh 208002, India

**Keywords:** Jailed wire, Rescue wire, Tangling technique, Wondering wire

## Abstract

A 38-year-old man underwent coronary angiography in our institution due to acute myocardial infarction as part of pharmaco-invasive strategy following thrombolysis. The patient showed total occlusion of mid left anterior descending (LAD) artery which was tortuous and calcified. The planned treatment was percutaneous coronary intervention (PCI) of culprit artery with wire being “jailed” in obtuse marginal branch of left circumflex artery (LCX) as left main was short and because of lesion characteristics. After successful stent implantation in the LAD, the “jailed” wire fractured as guiding catheter got deeply intubated as stent was being deployed in LAD. Initially, two balance middle weight (BMW) wires were used to retrieve but failed. Wire was wondering as it moved to proximal LCX, left main, partly into aortic sinus, sometimes proximal LAD and finally to LCX again during retrieval. Then it was decided to use tangling technique with the help of three BMW wires acting as rescue wires. The proximal ends of all three wires were inserted together in a torque device which were firmly screwed and rotated 40 - 50 times in circular pattern. During this rotational motion, the broken segment was tangled within these rescue wires and all four wires were removed together.

## Introduction

Virtually every coronary angioplasty device is advanced into the coronary system over a wire. The soft, atraumatic tips of coronary wires have been known to fracture off, if being manipulated excessively, and embolize in the coronary circulation. This most frequently occurs when the shapeable wire tip becomes lodged in an atheromatous plaque and separates from the body of the wire when the wire is retracted or entrapment, overcoiling and excessive traction of the guide wire can cause it to fracture. Because of manufacturing design, the incidence was a little high in the past but as current coronary wires are constructed of gradually tapering filaments, so relatively weak junction are minimized in contemporary wire design. Nevertheless it still occurs with incidence around 0.2-0.8% [1]. If the fragment of guide wire is not removed, there is a likelihood of acute embolization leading to acute coronary thrombus. Our case was caused by a deep intubation of guiding catheter into LAD as vessel was tortuous and long stent was being delivered leading to fracture of jailed wire of LCX. This type of complication is rarely reported as jailed wire kept wondering from proximal LCX, left main, partly into aortic sinus, sometimes proximal LAD and finally to LCX again during retrieval which was then successfully retrieved by tangling technique with the help of three BMW wires acting as rescue wires.

## Case Report

**Figure 1:**
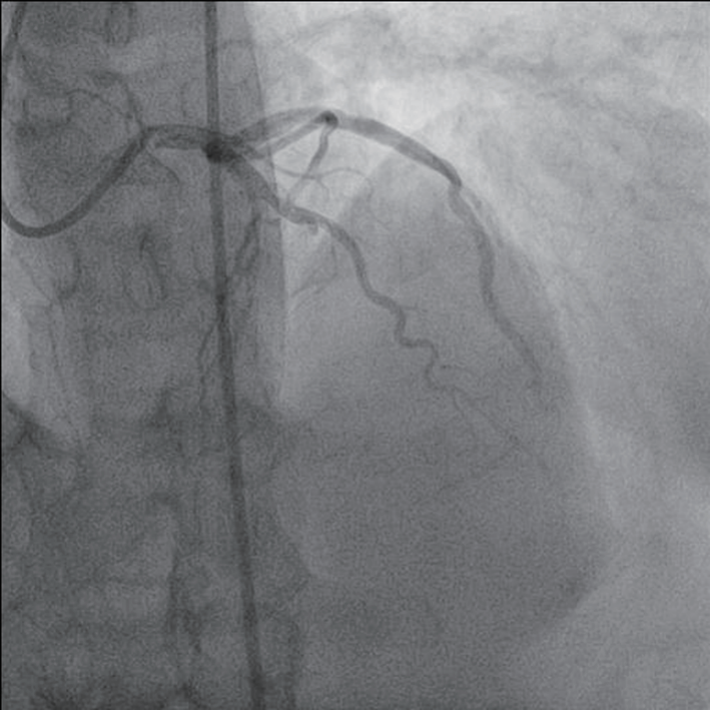
Antero-posterior (AP) cranial view showing total occlusion of mid left anterior descending (LAD) artery.

**Figure 2:**
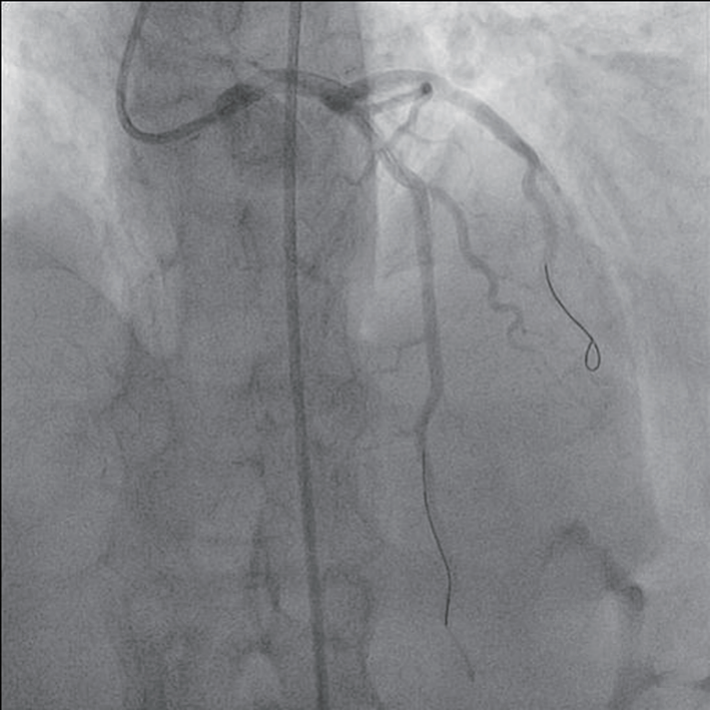
Antero-posterior (AP) cranial view showing post procedural TIMI III flow of left anterior descending (LAD) artery.

**Figure 3:**
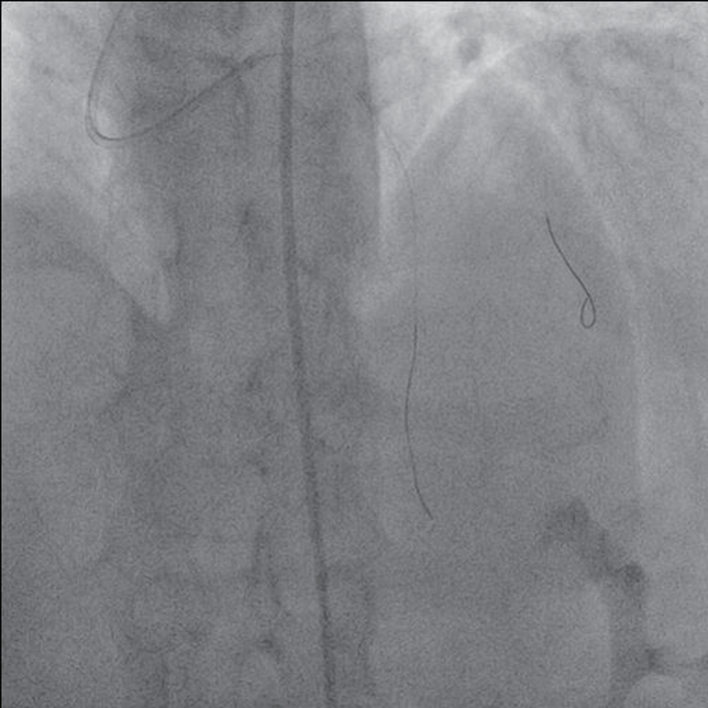
Antero-posterior (AP) cranial view showing broken distal segment of jailed wire in obtuse marginal branch of left circumflex artery (LCX).

**Figure 4:**
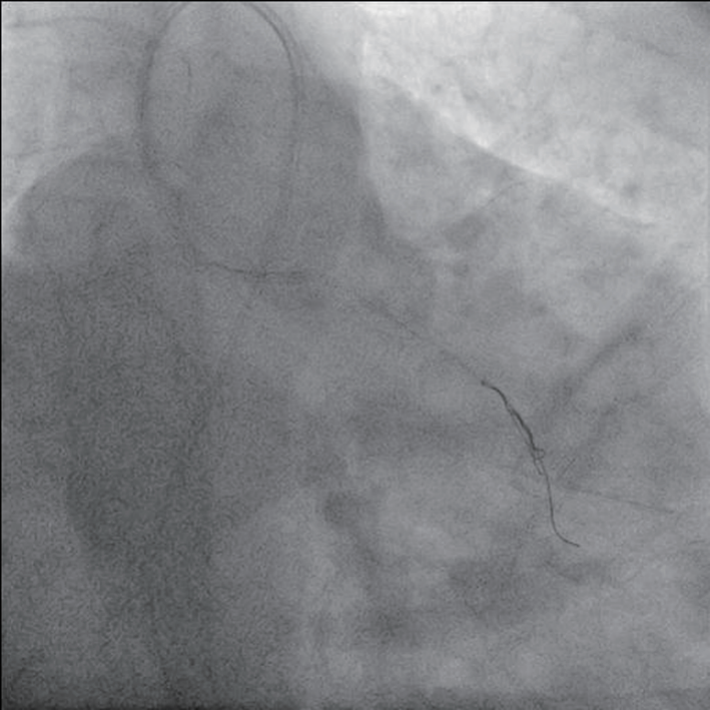
Antero-posterior (AP) caudal view showing distal segment of jailed wire being entangled by two BMW wires.

**Figure 5:**
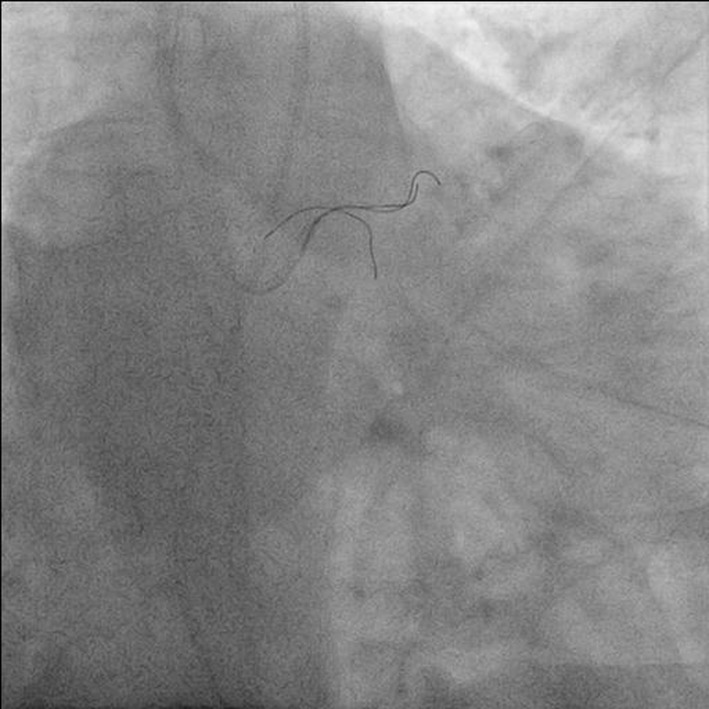
Antero-posterior (AP) caudal view showing one free end in aortic sinus and other end in proximal left circumflex artery (LCX).

**Figure 6:**
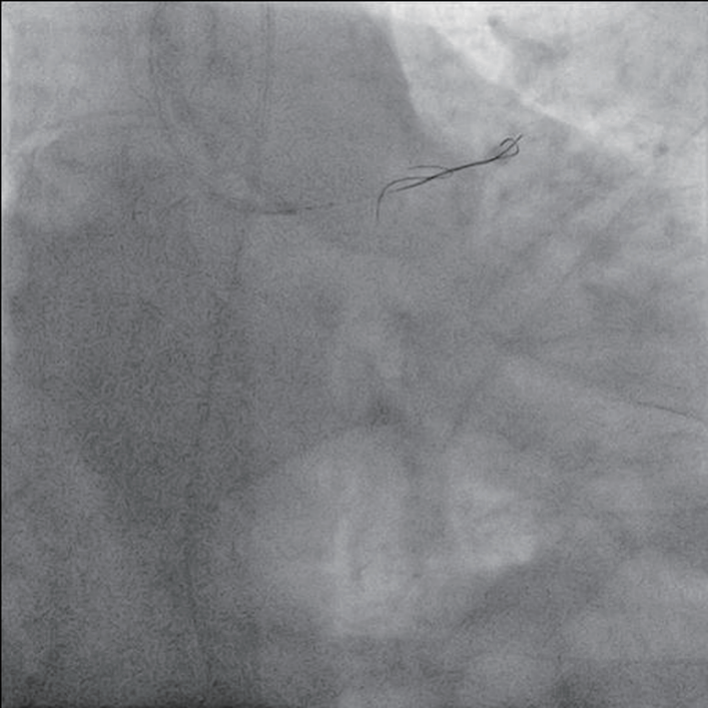
Antero-posterior (AP) caudal view showing broken wire in proximal left anterior descending (LAD) artery and other end in proximal LCX.

**Figure 7:**
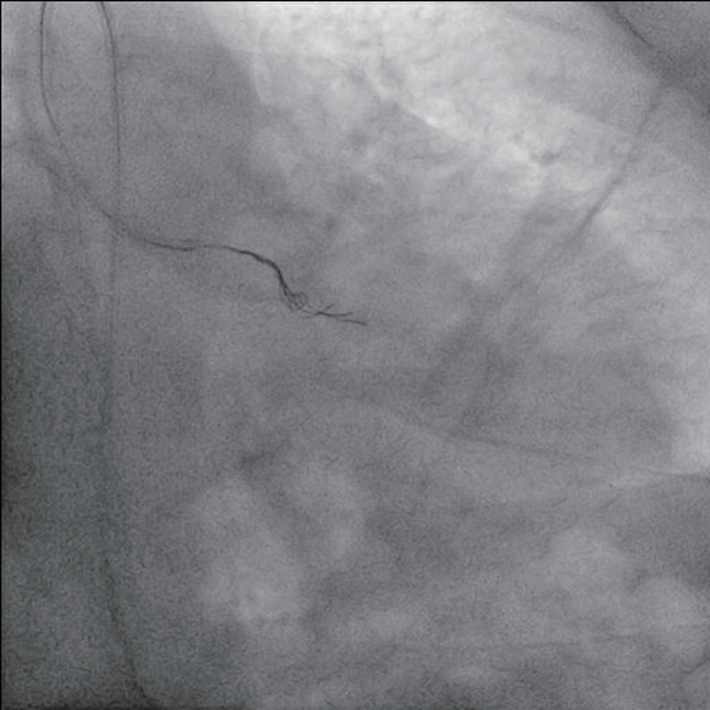
Antero-posterior (AP) caudal view showing distal segment of jailed wire being entangled by triple rescue wires.

A 38-year-old man with diabetes and hypertension of 2-year duration was admitted with acute anterior wall myocardial infarction for which he was thrombolyzed with 30 mg tenecteplase. He underwent coronary artery angiography on following day. PCI was planned because of total occlusion of mid LAD artery (Fig. 1). Intravenous heparin (100 U/kg) was administered. Left main was hooked with 6 F Launcher^®^ Coronary Guide Catheter (Medtronic, USA). Lesion was crossed by Fielder wire (Asahi Intecc, Japan) which was passed through FineCross^TM^ MG microcatheter (Terumo Medical Corp., NJ, USA) and parked distally. Wire was exchanged with 0.014ʺ 190 cm Hi-Torque BMW (Abbott Vascular, Santa Clara, USA) and microcatheter was removed by Nanto’s technique. Lesion was repeatedly dilated with 1.25 × 6, 1.5 × 10, 2 × 10 and 2.5 × 10 Sprinter Legend RX Balloon (Medtronic, USA). As artery appeared tortuous and lesion was calcified, it was planned to stent LAD with jailing the BMW wire into obtuse marginal branch of LCX to have better support and tracking of stent. Xience Prime 2.75 × 30 mm (Abbott Vascular, Santa Clara, USA) was deployed in distal LAD at 12 atm. Xience Prime 3 × 28 mm was deployed in proximal LAD overlapping with distal stent at 13 atm with post procedural TIMI III flow (Fig. 2). During proximal stent deployment, guiding catheter got deeply intubated causing fracture of “jailed” wire (Fig. 3). Two BMW wires were parked in distal LCX and proximal ends of both wires were passed through torque device and rotated 40 - 50 times to catch hold it wire (Fig. 4). While pulling it slipped in left main, one end was floating in aortic sinus and other was in proximal LCX (Fig. 5). Similar maneuver was applied but this time it migrated to proximal LAD (Fig. 6) and finally to distal LCX again. Then it was decided to use three BMW wires which were parked in distal LCX and proximal ends of all three wires were passed through torque device and rotated 40 - 50 times to catch hold it wire and all four wires were pulled inside guiding catheter and successfully retrieved (Fig. 7).

## Discussion

It is generally believed that the retained guide wire in the coronary tree may result in complications such as emboli, thrombosis, dissection and rupture and should be removed from the coronary system [2, 3]. Various solutions have been described to solve the problem of a broken guide wire in a coronary artery, ranging from surgery to simply leaving the segment of wire in place in cases where it could not be removed but first, efforts should be made to remove the guide wire percutaneously [4, 5]. However, it can be left as such in vessels that are already occluded or in very small branches. In our case as obtuse margin was quite large and LCX was dominant, we decided to retrieve it in any way. As snare loop wire was not available, simple technique was tried. Two wires were used first but it slipped in proximal LCX and then it migrated to left main, proximal LAD, once floating into aortic sinus and finally to obtuse marginal branch again. Finally tangling technique with the help of three wires acting as rescue wires was used to successfully retrieve the broken wire. Usually wire breaks in the artery which is being tackled for various reasons already described but our case was unique as jailed wire of LCX got broken by the guiding catheter at the time when proximal stent was being deployed in LAD. To the best of our knowledge, it is being reported for the first time.
